# Description of a new species of *Lamellothyrea* Krikken (Coleoptera, Scarabaeidae, Cetoniinae) from the iSimangaliso Wetland Park, KwaZulu-Natal (South Africa)

**DOI:** 10.3897/zookeys.688.13632

**Published:** 2017-08-08

**Authors:** Renzo Perissinotto

**Affiliations:** 1 School of Environmental Sciences, Nelson Mandela Metropolitan University, Summerstrand South Campus, P.O. Box 77000, Port Elizabeth 6031, South Africa

**Keywords:** *Lamellothyrea*, Cetoniinae, new species, South Africa, Mozambique

## Abstract

Recent data and material obtained from northern KwaZulu-Natal (South Africa) and Maputo Bay (Mozambique) have provided support for the description of a new species of the genus *Lamellothyrea* Krikken, 1980. The genus previously included only one species, *L. descarpentriesi*, with uncertain and poorly defined type locality, i.e. “Transvaal”. It is now evident that two different species are actually involved, *L. descarpentriesi* with currently known distribution limited to the coastal area north of Maputo, and *L. isimangaliso* **sp. n.** with a known distribution range virtually restricted to the iSimangaliso Wetland Park, in north-eastern KwaZulu-Natal. The two species appear to be separated by a substantial discontinuity in southern Mozambique and can be easily separated on the basis of their clypeal structure, extent of white dorsal tomentum and shape of aedeagal parameres. Both species appear to be restricted to the coastal belt, with *L. isimangaliso* sp. n. occupying almost exclusively dune forest habitats. In this species, adult activity depends on rainfall and shows two peaks, one at the onset of summer and the second in autumn.

## Introduction

The genus *Lamellothyrea* was described by Krikken in 1980 on the basis of a single male specimen reportedly from the “Transvaal” in South Africa. It has until now included only one species, *L. descarpentriesi*, with a reported distribution restricted to the coastal area of northern KwaZulu-Natal (KZN) and a vague type locality. It has now emerged, however, that two specimens recently collected along the Mozambique coast, north of Maputo Bay represent a different species to that occurring in northern KZN. An analysis of these specimens has revealed that they exhibit remarkable similarity with the holotype and conform well to the original description of Krikken (1980).

On the other hand, the numerous material now available for the KwaZulu-Natal population shows that this is distinct in many respects from the Mozambique and holotype specimens. This distinction appears to be best expressed at the level of the clypeal armour and in the parameres of the male genitalia. Thus, two separate species are involved, with the KwaZulu-Natal species here described as *L. isimangaliso* sp. n.

Substantial ecological data and observations have also become available from the recent studies undertaken in the iSimangaliso Wetland Park in KwaZulu-Natal, within its “Rare, Threatened and Endemic Species Project” (Combrink and Kyle 2006). This has now provided some key information on the distribution, relative abundance, habitat characteristics and general biology of the new species.

## Materials and methods

Since the original description of *Lamellothyrea* (Krikken 1980), numerous specimens from this genus have been collected in the iSimangaliso Wetland Park during the period leading up to, and following, its proclamation as UNESCO World Heritage Site (Combrink and Kyle 2006). Specimens were obtained mainly through deployment of aerial fruit-baited traps, inspection of flowers and fruits of waterberry trees (*Syzygium cordatum*) and direct search on the ground. Given the scarcity of museum/collection material, one observation record of *L. descarpentriesi* from Mozambique located on the citizen science platform iSpot (Silvertown et al. 2015) was also included in this study.

For the description of morphological characters, the terminology used by Krikken (1984) and Holm and Marais (1992) is followed in this study. Specimen total length and maximum width were measured using a Vernier calliper, from the anterior margin of the clypeus to the apex of the pygidium and at the widest point of the elytra, respectively. Photos of specimen dorsal and ventral habitus were taken with a Nikon CoolPix S9700 digital camera with macro setting, while photos of the male genitalia were obtained using a Nikon DigitalSight DS-Fi2 camera attached to a Nikon SMZ25 dissecting microscope. The background was removed from the photos using Microsoft Word 2010 (Picture Tools), in order to increase clarity of resolution. The Combine ZP Image Stacking Software by Alan Hadley (alan@micropics.org.uk) was used to obtain z-stacking composite images.

### Repositories are abbreviated as follows

**BMPC** Jonathan Ball and Andre Marais Private Collection, Cape Town, South Africa;

**CDPC** Cyril Di Gennaro Private Collection, Arcueil, France;

**DMPC** Daniel Moore Private Collection, Oro Valley, USA;

**DMSA** Durban Natural Science Museum, Durban, South Africa;

**EPPC** Ernest Pringle Private Collection, Bedford, South Africa;

**GBPC** Gerhard Beinhundner Private Collection, Euerbach, Germany;

**ISAM** Iziko South African Museum, Cape Town, South Africa;

**PCPC** Renzo Perissinotto and Lynette Clennell Private Collection, Port Elizabeth, South Africa;

**RMNH** Museum Naturalis, Leiden, The Netherlands;

**SANC** South African National Collection of Insects, Pretoria, South Africa;

**SRPC** Sébastien Rojkoff Private Collection, Lyon, France;

**TGPC** Thierry Garnier Private Collection, Montpellier, France;

**TMSA** Ditsong National Museum of Natural History (formerly Transvaal Museum), Pretoria, South Africa.

Data on distribution and period of adult activity were also obtained from the literature (Rigout and Allard 1992; Sakai and Nagai 1998) and the original description of *L. descarpentriesi* (Krikken 1980).

## Taxonomy

### Lamellothyrea descarpentriesi

Taxon classificationAnimaliaColeopteraScarabaeidae

Krikken, 1980

Lamellothyrea descarpentriesi Krikken, 1980: 185–187; Holm and Marais 1992: 126–127; Rigout and Allard 1992: 85, 92; Sakai and Nagai 1998: 332, 395.

#### Known material.

Holotype (♂): Transvaal (RMNH). Other material: 1♂, Mozambique, Marracuene 10 m, 50 km N of Maputo, S25°46'14", E32°40'11", 23–26 Aug 2001, AK Brinkman leg (BMPC); 1♀: Mozambique, Praia Do Bilene (25°28'63"S, 33°25'71"E) 27 Oct 2015, found dead on the beach, Andrew Deacon (https://www.ispotnature.org/node/747181, accessed on 25 May 2016).

#### Description of female.

**Figure 1.**
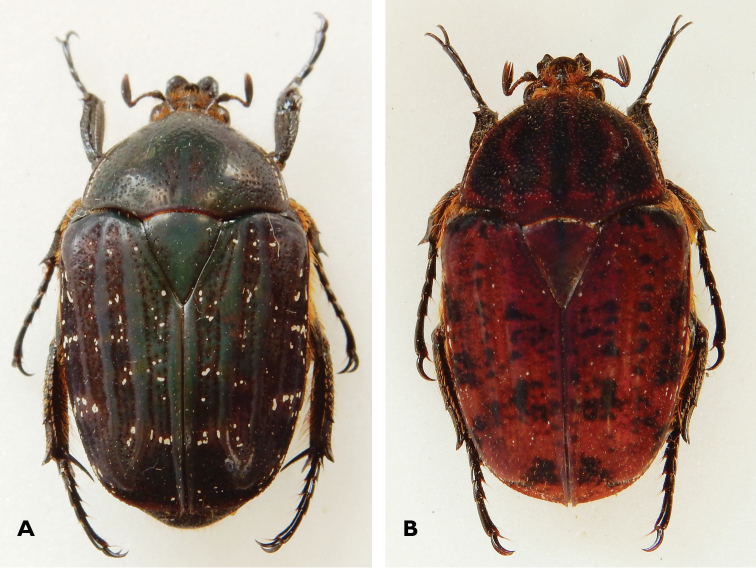
Dorsal habitus of **A** *Lamellothyrea descarpentriesi* (Marracuene, Mozambique) and **B** *Lamellothyrea isimangaliso* sp. n. (St Lucia, South Africa).

**Figure 2.**
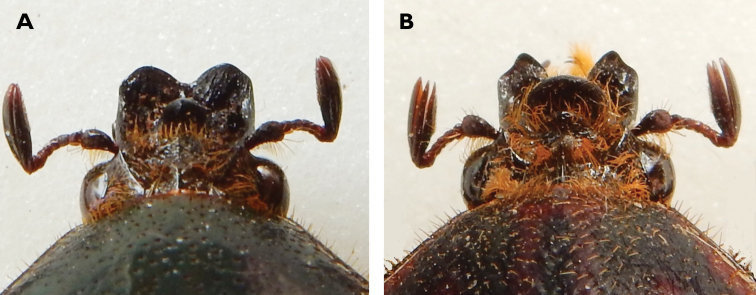
Clypeal armour of **A** *Lamellothyrea descarpentriesi* (Marracuene, Mozambique) and **B** *Lamellothyrea isimangaliso* sp. n. (St Lucia, South Africa).

While a detailed description of the holotype male of this species, complete with quality drawings, is provided in Krikken (1980), the female has remained unknown until the recent posting of a photo of a specimen by A. Deacon on the iSpot site. The specimen was found freshly dead on the beach of Praia do Bilene and the photo is of sufficiently high resolution to ascertain that it belongs to this sex and to recognise its key characters. On the basis of this, the female is essentially identical to the male in general appearance (Figs 1A, 2A), with the exception that it exhibits convex abdominal sternites without the medial vertical groove, which is typical of the male. The protibiae are also significantly broader anteriorly than in the male, and visibly tridentate.

### Lamellothyrea isimangaliso sp. n.

Taxon classificationAnimaliaColeopteraScarabaeidae

http://zoobank.org/B0BBF1C9-AFA0-4D89-80AA-5B17DC87A409

#### Diagnosis.

The two species of *Lamellothyrea* can easily be separated on the basis of their key differences at the level of the clypeal armour, parameres of the male genitalia and the general body colour and ornamentation. In particular, both clypeal horns and longitudinal blade are more pronounced and developed in *L. isimangaliso* sp. n. than in *L. descarpentriesi*. The clypeal horns of *L. isimangaliso* are narrower but projected further forward than those of *L. descarpentriesi*; they are also sharper and form a distinct point at the internal apex. The total body length of *L. descarpentriesi* appears to be slightly shorter than that of *L. isimangaliso* and the tomentose maculation on the dorsal surface is much more developed in the former than in the latter species. *Lamellothyrea descarpentriesi* is also more uniform in its background colouration, which is consistently dark green, while a purple sheen and even brown-green dominance generally prevails in *L. isimangaliso*. Finally, the aedeagal parameres are slightly longer in *L. isimangaliso*, with their dorsal lobes narrowing substantially in the middle and then forming a sinuate apex with a visible protuberance on each side. In *L. descarpentriesi*, the dorsal lobes are more compact and virtually lack both central constriction and apical protuberances.

#### Description of holotype male

**Figure 3.**
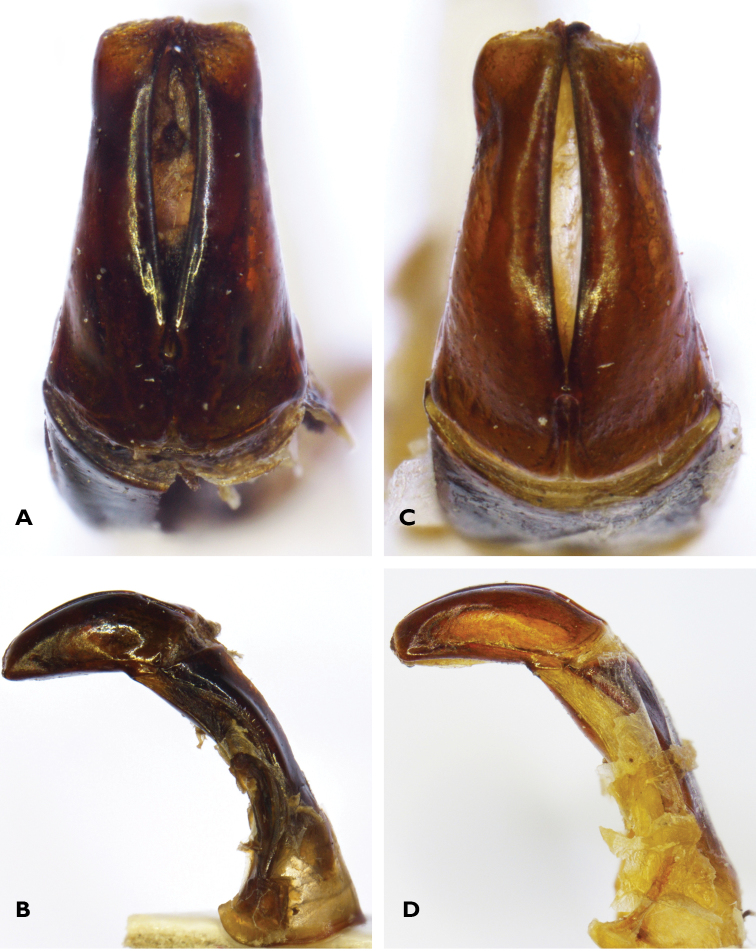
Dorsal (**A**) and lateral (**B**) views of aedeagal parameres of *Lamellothyrea descarpentriesi* (Marracuene, Mozambique); dorsal (**C**) and lateral (**D**) views of same for *Lamellothyrea isimangaliso* sp. n. (St Lucia, South Africa).

**Figure 4.**
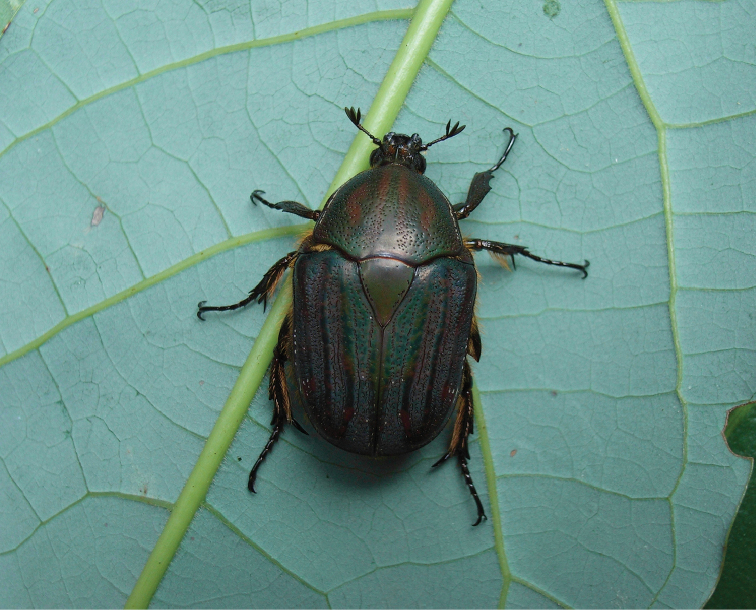
*Lamellothyrea isimangaliso* sp. n. in its natural habitat at St Lucia Estuary (Photo: Lynette Clennell, March 2009).

(Figs 1B, 2B, 3C–D, 4). *Size*. Length 20.3; width 11.0 mm.

*Body*. Dark green to purple, with blackish maculation on elytra and residual tomentum on pronotal sides and elytral surface (Figs 1B, 2B, 4); velutinous, with remarkable sheen when exposed to direct light (Fig. 4).

*Head*. Black to dark green towards vertex; clypeus deeply concave and sharply upturned at anterior margin, where two symmetrical lateral horns with external indentation project forward (Fig. 2B); frons with one major transverse round lamina, one minor frontal lamina and one longitudinal ridge extending to posterior end of vertex; numerous long, yellow to orange setae emerging from all cavities; frontal cavity forming secondary, shorter horns at lateral margins; entire surface covered in coarse, round punctures or geminate striae, except on clypeal ridges and supra-ocular tubercle (Figs 1B, 2B); antennal clubs dark brown, of normal cetonid length, of approximately the same length of flagellum; pedicel dark brown to black, flagellum brown, both bearing scattered but long, erected yellow setae.

*Pronotum*. Octagonal with angles smoothly rounded; lateral margins carinate and exhibiting white cretaceous band often interrupted, particularly near base and apex; posterior margin trisinuate with pre-scutellar arch marked; purple to brown, with widespread iridescence and five longitudinal black to dark-green bands (narrow one at center and two broader ones on each side); matt with dense crescent to semi-crescent sculpture, except on pre-scutellar arch; short, scattered yellow setae present throughout surface, longer at margins but absent on pre-scutellar arch (Figs 1B, 4).

*Scutellum.* Purple to brown and even black at base; generally smooth, with few scattered punctures and setae at margins only; broadly triangular with sharp apex and without lateral grooves (Fig. 1B).

*Elytron*. With moderately elevated sutural, discal, humeral and lateral costae; colour varying from purple to brown, with dark spots and bands particularly developed on apical half, on lateral declivity and above humeral callus; residual tomentose marking noticeable only along lateral declivity; both humeral and apical calluses pronounced; apical margin smoothly rounded, bearing a short proximal spine; crescent to horseshoe punctures on all interstrial surfaces, with very short yellow setae scattered throughout but becoming longer on lateral and apical declivities (Figs 1B, 4).

*Pygidium*. Triangular and remarkably convex; black to dark green, without any tomentum; with dense layered to wrinkled sculpture throughout and short yellow setae at centre, becoming much longer on apico-lateral margins.

*Legs*. Slender and elongate, with apical tarsal segments hypertrophic; protibia effectively bidentate, with third tooth obsolete and well-developed longitudinal grooves, with sparse short yellow setae, becoming longer and denser on inner margin; meso- and metatibia with longer and denser yellow setae, with striolate surfaces and mid spine on outer carina, distal margin bi- and tridentate respectively; spurs slender and sharply pointed, twice as long in metatibia than in mesotibia (Fig. 1B).

*Ventral surface*. Shiny dark brown to green; exhibiting small and sparse crescent sculpture throughout surface; with long and dense yellow setae throughout surface, except on metasternum, metafemora and abdominal sternites; mesometasternal lobe very round and broadly expanded anteriorly; abdominal sternites with visible concavity at centre.

*Aedeagus*. Parameres with blunt apex in both lateral and dorsal view (Fig. 3C); dorsal lobes narrower than ventral lobes and sharply constricted at mid length, then expanded again at apex; apex broad with sinuations and small sutural projections at centre (Fig. 3C–D).

*Derivatio nominis.* The name *L. isimangaliso* sp. n. reflects its known distribution range, which, with the exception of the southern locality of Lake Nhlabane, falls entirely within the iSimangaliso Wetland Park, South Africa’s first UNESCO World Heritage Site.

#### Description of female.

There is little sexual dimorphism in this species. The main difference lies in the female exhibiting a tridentate and more enlarged protibia than the male. The general body shape of the female also appears more globose than that of the male, particularly at the level of the abdominal sternites, which bulge out quite significantly to impart a convex shape. The length of the antennal clubs is slightly shorter than in the male. Finally, the female metatibial spurs are more blunt, concave and laterally expanded, assuming the shape of a typical fossorial organ used to burrow into the dune sand (Fig. 3B).

#### Distribution.

The species appears to be virtually restricted to the coastal dune forest of the iSimangaliso Wetland Park. The only known record outside the Park is from Lake Nhlabane, which is located about 10 km southeast of its southern boundary.

#### Conservation status.

Although there are indications that coastal dune mining maybe negatively impacting the population of *L. isimangaliso* at the southernmost end of its distribution range, there are currently no threats to the species, as its habitat within the iSimangaliso Wetland Park is entirely under statutory protection. The Park was proclaimed as South Africa’s first UNESCO World Heritage Site in 1999, and is particularly recognised for its exceptional biodiversity and as a center of endemism (Porter 2013).

#### Biology.

Although its larval stages remain unknown, *L. isimangaliso* is a typical coastal forest dweller. The period of adult activity mirrors the pattern of rainfall in the region, with a bimodal distribution and peaks in November-December and February-March. Both sexes have been repeatedly observed feeding on flowers and fruits of dune waterberry trees, *Sizygium cordatum* and have occasionally been trapped using aerial devices baited with fermenting fruits (Combrink and Kyle 2006).

#### Remarks.

Within the type series, the size ranges as follows: ♂ length 20.1–21.4 mm, width 11.0–11.4 mm (n = 21); ♀ length 21.2–21.8 mm, width 11.3–12.0 mm (n = 11).

There is substantial variability in the background colouration of the numerous specimens of *L. isimangaliso* sp. n. examined, ranging from light-brown to purple and dark-green. The velutinous purple sheen is, however, the most dominant chromatic form in both sexes. The residual tomentose spots on the elytral and pronotal margins are always poorly noticeable and can virtually disappear completely in some specimens. The vittate pronotum seems to be a consistent feature, but the longitudinal bands range in colour from black to dark-green and even brown. The pygidial background colour is invariably dark green, but occasionally it exhibits residual tomentum near the basal margin.

#### Type material examined.

Holotype (♂): South Africa, KZN, St Lucia, 19–20 Feb 2000, R Perissinotto & L Clennell (TMSA). Paratypes: 2♂♂, as above (PCPC); 1 ♀, as above, but 13 Jan 2001; 1♂, as above, but 17 Feb 2001 (PCPC); 1♀, as above, but 12–13 Feb 2000; 1♀, as above, but 01 Jan 1999 (PCPC); 2♂♂, as above, but 28 Feb 99 (PCPC); 1♂, as above, but 30 Oct 1999; 4♂♂, 4♀♀, as above, but 20–23 Mar 2004 (ISAM, DMSA); 1♂, but False Bay, 4 Apr 2012 (PCPC); 1♂, KZN, iSimangaliso, Dec 2002, X. Combrink leg (PCPC); 1♂, KZN, Kosi Mouth, 21/10/2003, R Kyle leg (PCPC); 1♂, Natal, Mapelane, 12/93, I.R. Willem (PCPC); 14♂♂ + 5♀♀, South Africa KwaZulu-Natal, 6 km N of St. Lucia Bay, S27°38'16", E32°26'24" 20 m a.s.l., 22–30 Dec 1990, P. Stobbia *leg.* (PCBM); 12♂♂ + 7♀♀, South Africa, KwaZulu-Natal, St. Lucia Bay S28°22'13", E32°24'50" 23 m a.s.l., 12 Dec 1994, A.P. Marais *leg.* (PCBM); 1 ♀, South Africa KZN, Sodwana Bay 27.33S, 32.40E 30 m, 1987/03/05, Reavell PE leg., Coastal dune forest, Feeding on fruit (unspecified) (SANC, COLS16846); 8♂♂ + 6♀♀, South Africa KZN, near Lake Nhlabane, 25 km NE of Richards Bay 28.38S 32.16E, 1991/02/03, Vogt M leg., Coastal dune forest, Hanging fermenting fruit bait trap, Specimen from University of Pretoria research programme into post-mining dune rehabilitation at Richards Bay Minerals, In rehabilitating dune forest 12 years post mining (SANC, COLS16847); 1♀, South Africa KZN, near Lake Nhlabane, 25 km NE of Richards Bay 28.38S, 32.16E, 1992/03/16 to 1992/03/20; Vogt M leg., Coastal dune forest, Hanging fermenting fruit bait trap, Specimen from University of Pretoria research programme into post-mining dune rehabilitation at Richards Bay Minerals (SANC, COLS16848); 1♂, South Africa KZN, Sodwana Bay 27.33S, 32.40E, 1988/01/05 to 1988-01-06, E Holm E & E Marais leg., Hanging fermenting fruit bait trap (SANC, COLS16849); 10 ind., South Africa, Zululand Natal, Sodwana Bay 5 km S, 27.35S, 32.39E, 23/11/1992, Endrody-Younga E-Y: 2845, Ground traps, 4 days, with banana bait (TMSA, CPH6477); 14 ind., South Africa, Zululand Natal, Sodwana Bay 5km S, 27.35S, 32.39E, 23/11/1992, Endrödy-Younga leg. E-Y: 2846, Hanging fruit traps (TMSA, CPH6478); 1 ind., South Africa, Zululand Natal, Sodwana Bay 5km S, 27.35S, 32.39E, 23/11/1992, Endrödy-Younga leg. E-Y: 2847, Grass netting (TMSA, CPH6479); 2 ind., South Africa, Natal, Sodwana Bay 27.32S, 32.42E, 16/1/1992, M. Vogt & E. Holm leg. (TMSA, CPH6480); 1 ind., South Africa, Natal, Sodwana Bay, 4–12–1988, HP Terblanche leg., Waterbessie (TMSA, CPH6481); 1 ind., South Africa, Zululand Natal, Sodwana Bay, 28/2/1982 (TMSA, CPH6482); 1 ind., South Africa, Natal, Sodwana Bay Nat. Res., 27.32S 32.4E, 1/1988, O. Bourquin leg., Baited forest trap (TMSA, CPH6483); 1 ind., South Africa, Natal, Sodwana Bay Nat. Park Cmpgrd., 9–13/11/1986, Evans, d’Hotman & Nel leg. (TMSA, CPH6484); 1 ind., South Africa, Natal, Cape Vidal Park Cmpgrd., 28.07S, 32.33E, 13–15/11/1986, Evans, d’Hotman & Nel leg. (TMSA, CPH6485); 1 ind., South Africa, Natal, Cape Vidal, St. Lucia Nat. Res., 28.08S 32.33E, 1–4/1/1988, E. Holm & E. Marais leg., Bait traps (TMSA, CPH6486); 2 ind., South Africa, KZN, St Lucia, 22 Mar 2004, R Perissinotto & L Clennell (DMSA, COL35760, COL35760); 1 ind., as above but 26 Oct 2004 (DMSA, COL35762); 3 ind., as above but 1 Oct 2008 (DMSA, COL35763, COL35764, COL35765); 2♂♂ + 1♀, RSA KwaZulu-Natal, St Lucia, 20–22/03/2004, Perissinotto & Clennell (TGPC); 2♂♂ + 2♀♀, RSA N. Natal, Sodwana Bay, 03/1994 (TGPC); 3♂♂ + 2♀♀, South Africa KZN, St Lucia, 20–22 Mar 2004, R Perissinotto & L Clennell (CDPC); 3 ind., South Africa, KwaZulu-Natal, St. Lucia –28.37.500S, 32.42694E, 20–22/3/1904 (most likely 2004), (ISAM, COL-A059118); 3♂♂ + 2♀♀, S-Africa Natal, Sta Lucia, 1–91, Ex collection Dr. Vincent Allard received from Christophe Allard 21.II.2015 (GBPC); 1♂, R.S.A. Zululand, Sodwana Bay, II-1987. P. Stobbia, Ex collection Dr. Vincent Allard received from Christophe Allard 21.II.2015 (GBPC); 1♀, R.S.A. Zululand, Sodwana Bay, 7–XI–1987. P. Stobbia, Ex collection Dr. Vincent Allard received from Christophe Allard 21.II.2015 (GBPC); 1♂ + 1♀ , RSA Natal, St. Lucia, 1.1991, coll. V. Allard (GBPC); 1♂ + 1♀, No data, Ex collection Dr. Vincent Allard received from Christophe Allard 21.II.2015 (GBPC); 1♀, RSA Natal, St. Lucia, 3.1992, coll. Owen (GBPC); 1♂, South Africa, , KZN, St Lucia 31 Oct 2008, R. Perissinotto & L. Clennell leg. (SRPC); 1♀, 11–XII–2008, South Africa, KZN, 11 Dec 2008, St Lucia R. Perissinotto & L. Clennell leg. (SRPC).

**Figure 5.**
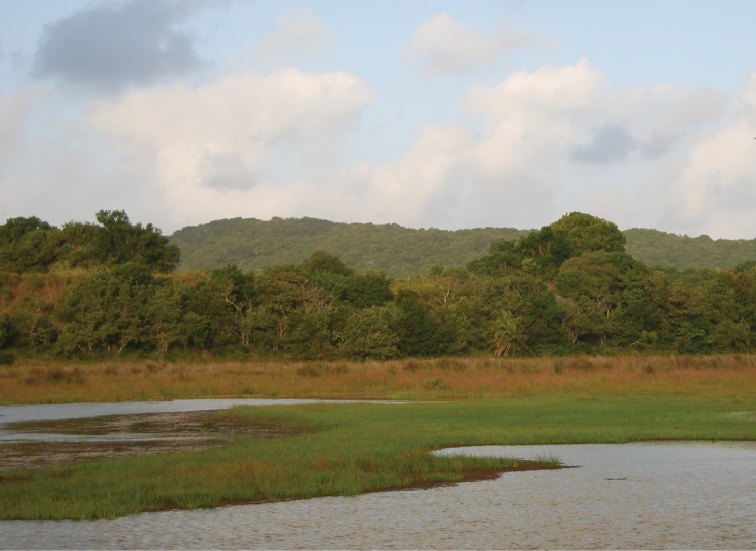
Typical habitat of *Lamellothyrea isimangaliso* sp. n. in the coastal forest on the Eastern Shores of Lake St Lucia (Photo: Nicola Carrasco, May 2010).

**Figure 6.**
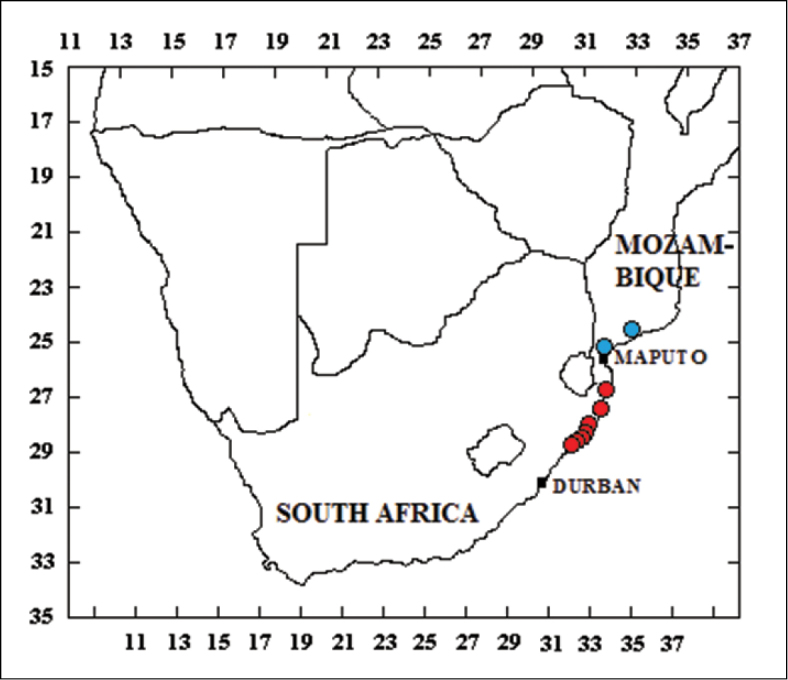
Known distribution of *Lamellothyrea descarpentriesi* (blue circles) and *L. isimangaliso* sp. n. (red circles).

## Discussion

Holm and Marais (1992) interpreted the different clypeal horn exhibited by the type specimen of *Lamellothyrea descarpentriesi* (used by Krikken in his description), compared to the other specimens from KwaZulu-Natal (KZN), as a result of wearing off of the apical angles. In the absence of other specimens of the true *L. descarpentriesi*, presumably they believed that the type specimen was an old individual, while all other specimens represented freshly emerged beetles. However, the recent availability of a further fresh specimen and photographs posted on the citizen science platform iSpot (Silvertown et al. 2015) of the true *L. descarpentriesi* have allowed a somehow unexpected resolution to their interpretation. Two separate species are obviously involved in this genus, with one distributed in Mozambique in an area of unknown extension to the north of Maputo Bay, and the other restricted to the Maputaland coast of KZN, from Kosi Bay in the north to Lake Nhlabane in the south. In fact, with the exception of the latter locality, which falls a few kms outside the borders of the iSimangaliso Wetland Park, the entire range of *L. isimangaliso* sp. n. is essentially inside the park itself. All specimens reported in the illustrations contained in Holm and Marais (1992), Rigout and Allard (1992), and Sakai and Nagai (1998) belong unequivocally in *L. isimangaliso*. Interestingly, the known distribution of the leaf chafer species *Asthenopholis rex* Harrison, 2009 and *A. adspersa* (Boheman 1857) is almost identical to this, and they are also coastal dune endemics (Harrison 2009).

The relatively short gap in distribution between the two species, i.e. about 130 km, is surprising to some extent, but actually involves a major climatic and vegetation transition from wetter coastal forests in the south to the drier swamp forests and Indian Ocean coastal belt vegetation in the north-east (Mucina and Rutherford 2006). Indeed, records of *L. isimangaliso* to the north of the coastal forests around Sodwana Bay and Lake Sibayi are few, and Kosi Bay itself has only one confirmed record thus far. On the other hand, the distance between the South African border inland of Maputo (formerly the Transvaal Province) to the Mozambican capital city itself is only about 60 km. Thus, it is possible that the vague locality of “Transvaal” associated with the holotype described by Krikken (1980) may represent a simple mislabelling error by a collector travelling from the South African province to the popular holiday destinations on the Mozambique coast. It is also plausible though that the holotype specimen may represent an unusual struggler occurrence of a specimen on a random dispersal trajectory.

While the only two dated records available for *L. descarpentriesi* show that this species is active at least from late winter (August) to early spring (October), in northern KZN adults of *L. isimangaliso* appear to be especially active during the rainy months, from October through May. In accordance with what was previously reported by Holm and Marais (1992), this species shows a population peak in November/December. However, records suggest it flies well beyond February, actually reaching a second lower peak in abundance around March. This follows the pattern of rainfall along the north coast of KwaZulu-Natal, with summer rains beginning in late September to early October, escalating in November, then subsiding in December/January to increase again in February/March and eventually ending in May.

Unfortunately, virtually nothing is yet known about the biology/ecology of *L. descarpentriesi*, as no other information is provided on the data labels that accompany the few specimens that are currently known. On the other hand, *L. isimangaliso* shows a preference for dense forest clumps and the immediate outskirts of forests. It has mostly been collected using fruit-baited traps containing either fermenting banana, pineapple, or a fruit-wine-sugar mixture. It has also been observed and captured hovering above, and feeding on, fruits and flowers of *Syzigium cordatum* (waterberry tree) and on sap of *Ziziphus* sp. (cf. specimen data labels). This species has so far only been observed in a narrow coastal forest belt, and appears to avoid moving further inland. This restricted distribution suggests that it is linked to a very specific microclimate and possibly able to tolerate only a narrow range of environmental variability. In this context, the protection afforded by the special status of the iSimangaliso Wetland Park, as an UNESCO World Heritage Site, is crucial towards the survival of this species in the long term.

## Supplementary Material

XML Treatment for Lamellothyrea descarpentriesi

XML Treatment for Lamellothyrea isimangaliso
